# Deep learning prediction of error and skill in robotic prostatectomy suturing

**DOI:** 10.1007/s00464-024-11341-5

**Published:** 2024-10-21

**Authors:** N. Sirajudeen, M. Boal, D. Anastasiou, J. Xu, D. Stoyanov, J. Kelly, J. W. Collins, A. Sridhar, E. Mazomenos, N. K. Francis

**Affiliations:** 1 Wellcome/ESPRC Centre for Interventional Surgical Sciences (WEISS), University College London (UCL), London, UK; 2 The Griffin Institute, Northwick Park and St Marks Hospital, London, UK; 3 Division of Surgery and Interventional Science, Research Department of Targeted Intervention, UCL, London, UK; 4 Medical Physics and Biomedical Engineering, UCL, London, UK; 5 Computer Vision, UCL, London, UK; 6 University College London Hospitals NHS Foundation Trust, London, UK; 7 Yeovil District Hospital, Somerset Foundation NHS Trust, Yeovil, UK

**Keywords:** Robotic, Deep learning, Errors, Technical skill

## Abstract

**Background:**

Manual objective assessment of skill and errors in minimally invasive surgery have been validated with correlation to surgical expertise and patient outcomes. However, assessment and error annotation can be subjective and are time-consuming processes, often precluding their use. Recent years have seen the development of artificial intelligence models to work towards automating the process to allow reduction of errors and truly objective assessment. This study aimed to validate surgical skill rating and error annotations in suturing gestures to inform the development and evaluation of AI models.

**Methods:**

SAR-RARP50 open data set was blindly, independently annotated at the gesture level in Robotic-Assisted Radical Prostatectomy (RARP) suturing. Manual objective assessment tools and error annotation methodology, Objective Clinical Human Reliability Analysis (OCHRA), were used as ground truth to train and test vision-based deep learning methods to estimate skill and errors. Analysis included descriptive statistics plus tool validity and reliability.

**Results:**

Fifty-four RARP videos (266 min) were analysed. Strong/excellent inter-rater reliability (range *r* = 0.70–0.89, *p* < 0.001) and very strong correlation (*r* = 0.92, *p* < 0.001) between objective assessment tools was demonstrated. Skill estimation of OSATS and M-GEARS had a Spearman’s Correlation Coefficient 0.37 and 0.36, respectively, with normalised mean absolute error representing a prediction error of 17.92% (inverted “accuracy” 82.08%) and 20.6% (inverted “accuracy” 79.4%) respectively. The best performing models in error prediction achieved mean absolute precision of 37.14%, area under the curve 65.10% and Macro-F1 58.97%.

**Conclusions:**

This is the first study to employ detailed error detection methodology and deep learning models within real robotic surgical video. This benchmark evaluation of AI models sets a foundation and promising approach for future advancements in automated technical skill assessment.

**Graphical abstract:**

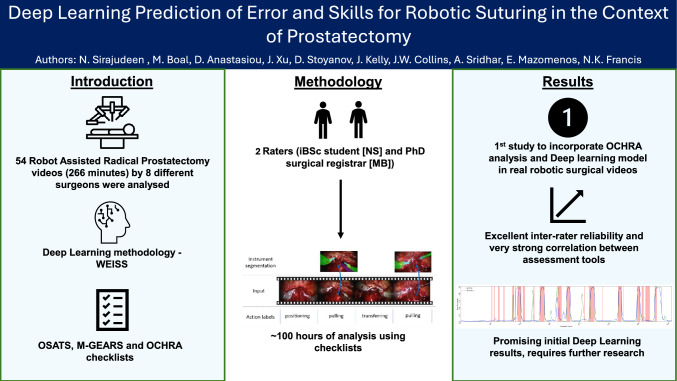

**Supplementary Information:**

The online version contains supplementary material available at 10.1007/s00464-024-11341-5.

Prostate cancer is the second most common form of cancer in men worldwide [1]. Globally, there was an estimated 1.4 million incident cases and 375,000 deaths in 2020 alone [2]. Robotic-assisted radical prostatectomy (RARP) is the gold standard surgical approach with improved morbidity compared to open [3, 4], with improved cost effectiveness and Quality Adjusted Life Years when compared to laparoscopy [5].

Traditionally, assessment and credentialing of a surgeon is determined by case number. There has been a shift nowadays, focusing on objective, proficiency-based progression [6], supported by evidence demonstrating reduction of procedural errors by up to 60% [7]. Studies have demonstrated that intra-operative assessment of technical skill and errors can be directly linked to patient outcome [8–10].

However, whilst improving surgical skill has great potential to positively influence clinical outcomes, drawbacks remain which prevent it from being integrated into current clinical practice. Manual assessment tools lack in full evaluation and scope and can be seen as subjective with wide variability in rater reliability [11]. Other issues include the time-consuming process of retrospective video analysis, with expert clinicians’ analyses impeded by busy clinical commitments.

There is a need for automated skill and error recognition, that provides truly objective assessment and augments surgeons’ performance enabling safer operating.

In recent years artificial intelligence (AI) models have been shown able to recognise and classify surgical activity down to granular surgical movements (gestures) [12]. Conversely, limited work has focused on analysing intraoperative data and automated performance metrics to predict skill and clinical outcomes [13–15]. Present approaches mostly focus on binary evaluation, of proper/improper execution of individual gestures [16–18]. No AI methods have evaluated error prediction within the clinical setting.

This study aimed to validate surgical skill rating and error annotations in suturing gestures to inform the development and evaluation of vision-based deep learning models.

## Materials and methods

Independent, blinded, frame-by-frame video review, using open-source software (VirtualDub2, https://www.virtualdub.org/), was performed by two raters, who analysed robotic suturing of the dorsal venous complex in RARP. Videos were analysed from an open-source dataset (SAR-RARP50 [19]). Each video had been previously annotated [12] with suturing gestures (Supplementary Table 1), acting as a task analysis sheet. Raters went through training consensus sessions to improve reliability, utilising suturing videos from the JIGSAWS dataset [20].

To objectively assess skill, two validated global rating scale tools were utilised: Objective Structured Assessment of Technical Skills (OSATS) (Supplementary Fig. 1), previously used on the JIGSAWS dataset, and Modifiable-Global Evaluative Assessment of Robotic Skills (M-GEARS) (Supplementary Fig. 2), a robotic-specific assessment tool. The total score available for both was 30 once domains, which were not applicable, were excluded.

For error annotation, a bespoke tool was developed from a validated suturing checklist [21] and methodology based on Observational Clinical Human Reliability Analysis (OCHRA) [18], a previously validated tool in minimally invasive surgery [9, 22–24]. It provided a list of possible procedural and executional errors within any given suturing task (Supplementary Table 2). Errors were annotated with a start and end frame within the gesture(s) they occurred, indicating the enactment and the end of an error including the recovery mechanism.

### Statistical analysis

Inter-rater reliability (IRR) of assessment tools scoring and ranks were analysed using Spearman’s (rho) and Pearson’s correlation (r) for the JIGSAWS and SAR-RARP50 datasets, respectively. Strength of correlation analysis was based on previously published analysis. Bland-Altmann plots were also produced to compare the agreement between raters; raters 1 and 2 for the SAR-RARP50 dataset, and an additional published rater 3 from the original JIGSAWS dataset. The research group defined an acceptable average difference of agreement to be ± 2 for OSATS and M-GEARS and a difference of ± 5 for OCHRA. Descriptive statistical analyses were used for error annotations. Concurrent validity between M-GEARS and OSATS was assessed using Pearson’s correlation coefficient. Kruskal–Wallis ANOVA tests were conducted to determine if the OSATS, M-GEARS and OCHRA scores correlated with the seniority level of the surgeon, to establish construct validity between three groups (junior registrar, senior registrar/early consultant and senior consultant) (Supplementary Table 3).

A repeated samples ANOVA test was conducted to determine variability in the total number of errors between the gestures, whilst a Chi-squared test was carried out to determine if the error type was more likely in certain gestures. All statistical methods employed were advised and results ratified by a university statistician.

### Deep learning methodology

Skill estimation employs a model comprising a Vision Transformer (ViT) [25] pretrained on the ImageNet dataset, trained in a self-supervised manner using DINOv2 [26], along with a Temporal Convolutional Network (TCN). To evaluate the model the dataset was portioned into 80% training and 20% testing. Within the training data, fourfold cross-validation was utilised to optimize hyperparameters. Subsequently, the network was retrained on the entire training set and its performance was assessed on the unseen test set. The evaluation relied on widely used metrics for skill score estimation, specifically the Spearman Correlation Coefficient (SCC) and the Mean Absolute Error (MAE).

Error detection employed five different vision-based deep learning models, namely ResNet-50 [27], TeCNO [28], MS-TCN [29], MS-TCN++  [30] and Asformer [31] to classify surgical video frames as normal or error frames. Three standard and commonly used metrics were used to evaluated model performance, mean Average Precision (mAP), Area Under Curve (AUC), and Macro-F1.

## Results

Fifty-four RARP videos, totalling 266 min and 10 s, from 8 surgeons were independently analysed with OSATS, M-GEARS and OCHRA, annotating 2507 errors. There was an estimated 100 h of analysis by one rater conducted for the skill and error annotation process.

### Rater training results on JIGSAWS

Inter-rater reliability analysis was performed on 10 videos in the JIGSAWS dataset. Strong to very strong IRR was demonstrated on the JIGSAWS dataset between the two raters in both the OSATS (rho = 0.954, 95% CI [0.806, 0.990], *p* < 0.001) and M-GEARS (rho = 0.841, 95% CI [0.433, 0.963], *p* = 0.002) scoring systems. Strong to very strong IRR was achieved between the OSATS score from rater 3 of the published JIGSAWS scores with rater 1 (rho = 0.809, 95% CI [0.345, 0.955], *p* = 0.005) and rater 2 (rho = 0.867, 95% CI [0.506, 0.969], *p* = 0.001).

Bland–Altman analysis demonstrated an average difference of 2.1 was found between rater 1 and rater 2, whilst average differences of 1.6 and 0.5 were found between the published rankings and raters 1 and 2 respectively using the OSATS scoring system. For M-GEARS, an average difference of 1.8 was found between rater 1 and rater 2.

There was a very strong correlation between OSATS and M-GEARS scores for both rater 1 (rho = 0.994, 95% CI [0.972, 0.999], *p* < 0.001) and rater 2 (rho = 0.869, 95% CI [0.514–0.970], *p* < 0.001).

### SAR-RARP50 results

Inter-rater reliability analysis was performed on 20 videos in the RARP dataset. There was strong IRR for OSATS scores (*r* = 0.7835, 95% CI [0.5219, 0.9104], *p* < 0.001) and ranks (*r* = 0.7609, 95% CI [0.48, 0.9003], *p* < 0.001). Strong IRR was demonstrated for M-GEARS scores (*r* = 0.7026, 95% CI [0.3774, 0.8735], *p* < 0.001) and ranks (*r* = 0.7281, 95% CI [0.4214, 0.8854], *p* < 0.001). Combining JISGAWS and SARS-RARP50 dataset scores demonstrated very strong IRR for OSATS (*r* = 0.894, 95% CI [0.7873, 0.9487] *p* < 0.001) and strong IRR for M-GEARS (*r* = 0.7938, 95% CI [0.6071, 0.8974], *p* < 0.001). Objective Clinical Human Reliability Analysis (OCHRA) demonstrated very strong IRR (*r* = 0.8726, 95% CI [0.6641, 0.9551], *p* < 0.001).

Bland–Altman analysis found an average difference 2.3 and 0.32 between rater 1 and rater 2 for OSATS and M-GEARS, respectively. Analysis for OCHRA results demonstrated an average number of errors at 46.43 per video, and an average difference of 5.87 errors (12.6% difference) between rater 1 and 2.

Very strong correlation of M-GEARS and OSATS was demonstrated for scores (*r* = 0.9227, 95% CI [0.8699, 0.9546], *p* < 0.001) and ranks (*r* = 0.9047, 95% CI [0.8406, 0.9438], *p* < 0.001). A moderate, statistically significant inverse correlation was established between OSATS and OCHRA number of errors (*r* = − 0.443, *p* = 0.000795) and rank correlation of (*r* = − 0.4159, *p* = 0.001762). M-GEARS similarly demonstrated a moderate to strong, negative correlation with scores (*r* = − 0.5265, *p* = 0.000042) and rankings (*r* = − 0.5262, *p* = 0.000044).

There was no significant difference in M-GEARS (*p* = 0.483), OSATS (*p* = 0.207) and OCHRA (*p* = 0.691) scores comparing seniority groups using the Kruskall Wallis ANOVA test. Pairwise comparison showed no significant differences between groups (*p* = 0.723–0.999).

### Error analysis

The ‘Pulling the needle out of the tissue’ gesture contained the greatest number of errors (*n* = 726) (Table 1). ‘Tying the Knot’ was the next most erroneous gesture (*n* = 696), whilst ‘Cutting the Suture’ gesture contained the least [19]. Error category ‘Incorrect/poor instrument control’ occurred the most frequently (*n* = 686) followed by ‘Multiple attempts’ (*n* = 510). The mode for errors and gestures was ‘Needle out of view’ with ‘Tying the Knot’ (*n* = 249), followed by ‘Incorrect/poor instrument control’ with ‘Pushing needle through tissue’ (*n* = 200). Many errors did not occur within any gesture. Analysis showed a statistically significant difference in the frequency of each error type (*χ*^2^ = 1945.15, *p* < 2.2e-16), some errors were more common in certain gestures (*p* = 0.0004998). The repeated samples ANOVA test found a statistically significant difference in the number of errors performed between the different gestures (F-statistic = 74.7987, *p* = 0). No statistically significant difference was found between gesture 1 (picking up needle) and gesture 2 (positioning needle tip/positioning needle in needle driver), between gestures 3 (pushing needle through tissue), 4 (pulling needle out of tissue) and 5 (tying the knot) and between gestures 6 (cutting the suture), 7 (returning/dropping the needle) and 8 (other).

**Table 1:** Number of errors for each gesture

Error type	Gesture type								
	Picking up needle	Positioning needle tip/ positioning needle in the needle driver	Pushing needle through tissue	Pulling needle out of tissue	Tying the Knot	Cutting the suture	Returning/dropping the needle	Other gestures in suturing	Total
Multiple attempts	9	49	127	194	92	6	13	20	510
Needle drop/slip if in tissue	1	6	10	40	19	0	0	1	77
Instruments out of view	0	0	0	1	0	0	0	0	1
Needle out of view	4	0	3	0	249	0	1	2	259
Tissue damage	13	13	23	44	12	2	2	4	113
Incorrect angle grasping needle	0	1	78	22	15	0	0	0	116
Incorrect position along needle	1	6	71	6	5	0	0	0	89
Excessive force	0	5	6	28	19	2	0	5	65
Needle does not follow the curve	0	0	1	104	64	0	0	0	169
Needle entry incorrect angle	0	0	10	25	0	0	0	0	35
Grasped at needle tip	0	1	10	88	7	0	0	0	106
Thread caught in instrument	3	5	23	18	17	0	0	1	67
Inadequate no of throws	0	0	0	0	0	1	0	0	1
Incorrect distancing between needle drives	0	0	1	3	0	0	0	0	4
Suture not pulled through between needle drives	0	0	1	0	3	0	0	0	4
Suture entanglement	0	7	16	3	7	1	0	0	34
Fraying the suture	0	0	0	0	3	2	0	0	5
Snapping the suture	0	1	0	0	3	0	0	0	4
Incorrect/poor camera control	19	9	20	15	85	1	5	8	162
Incorrect/poor instrument control	111	67	200	135	96	4	38	35	686
Total	161	170	600	726	696	19	59	76	2507

### Deep learning results

Skill estimation (Table 2) of OSATS and GEARS had a Spearman’s Correlation Coefficient 0.37 and 0.36, respectively, with normalised mean absolute error representing a prediction error of 17.92% (inverted “accuracy” 82.08%) and 20.6% (inverted “accuracy” 79.4%) respectively.

**Table 2:** Skill estimation on the SAR-RARP50 dataset

OSATS		M-GEARS	
SCC	MAE	SCC	MAE
0.37	2.15	0.26	2.06

**Fig. 1:**
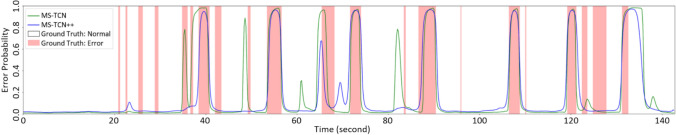
Visualization of the error detection results of MS-TCN and MS-TCN++ on the SAR-RARP50 dataset. The x-axis represents the time and the y-axis is the error probability output by the models. Error frames are depicted with a red background, whilst normal frames are depicted with a white background (Color figure online)

**Fig. 2:**
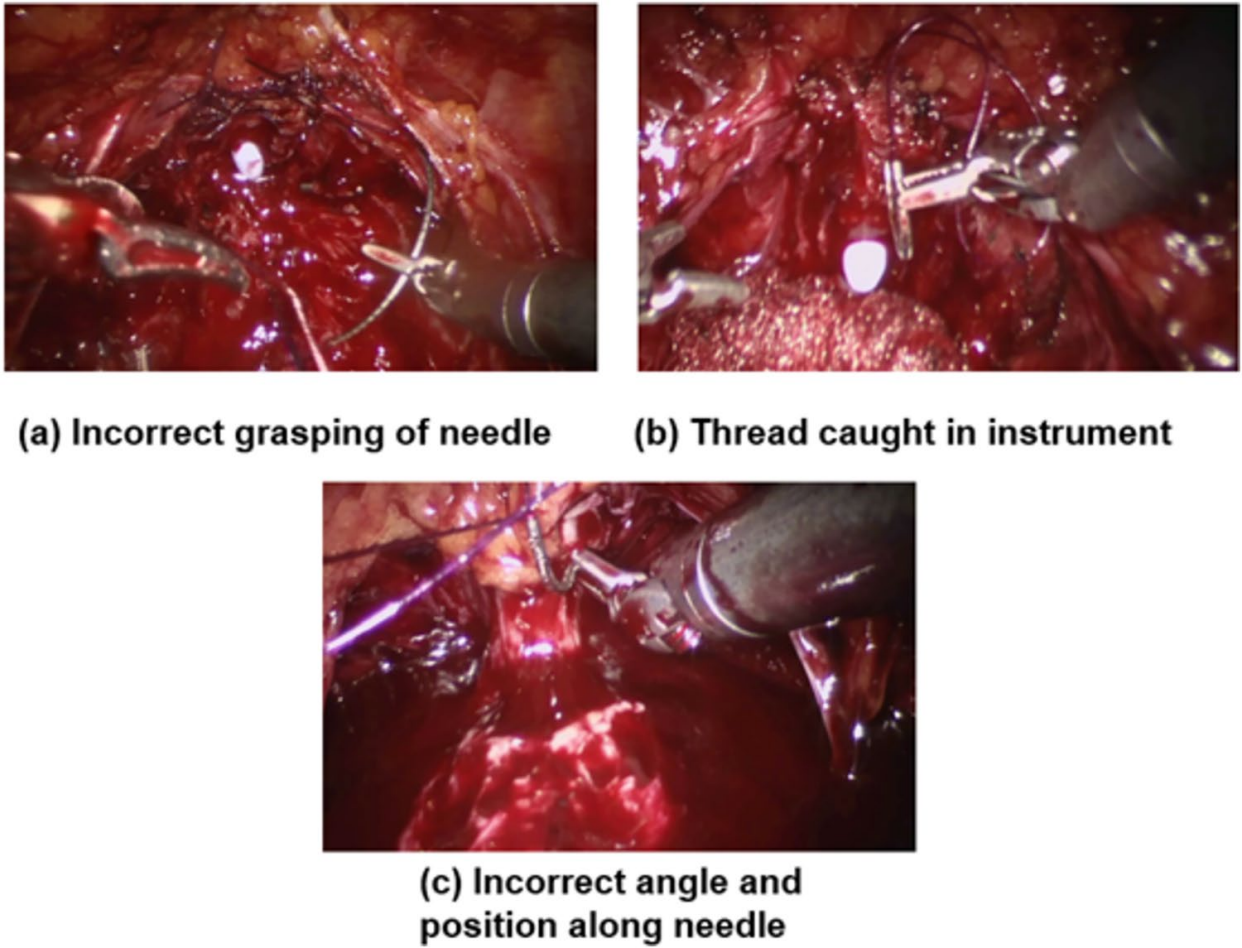
Examples of commonly annotated errors of the SAR-RARP50 dataset

**Fig. 3:**
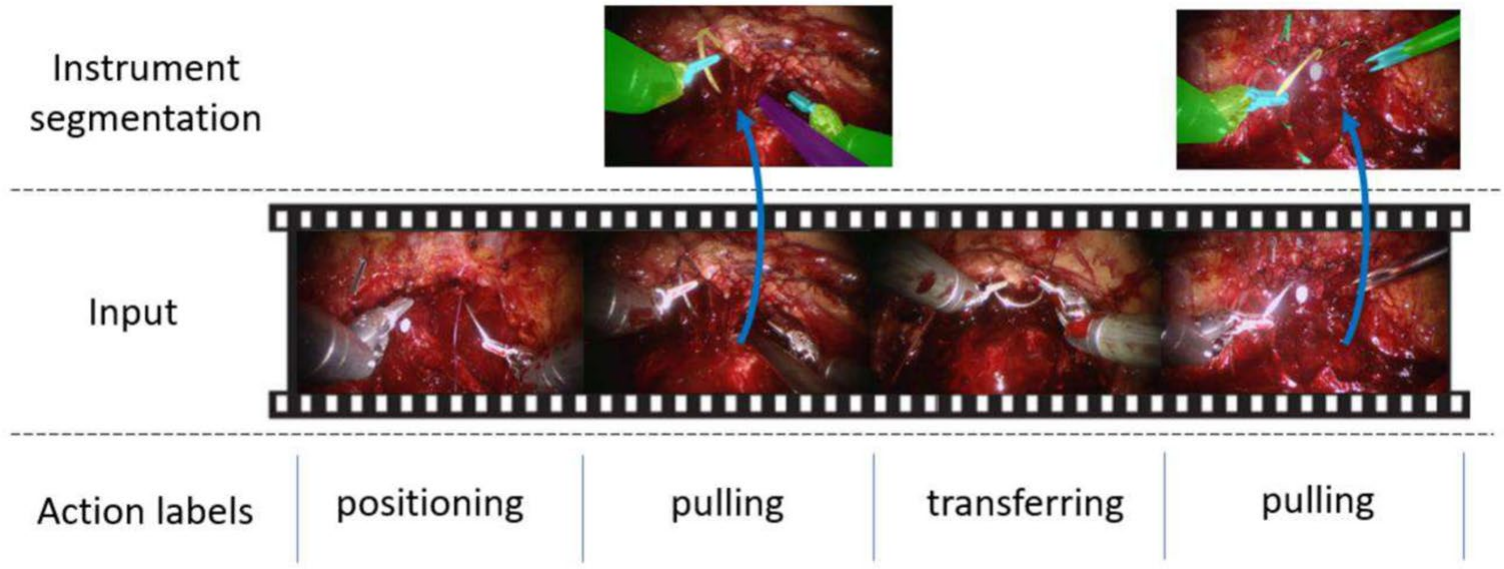
Gesture examples within the RARP-50 dataset

Error estimation (Table 3) illustrates that MS-TCN++ achieved the highest AUC and Macro-F1 with scores of 65.10% and 58.97%, respectively. Its variant, MS-TCN, demonstrated the highest mAP score of 37.14%. Moreover, we present the error detection outputs of the top two models, MS-TCN++ and MS-TCN, in Fig. 1. Both models showcased superior performance in detecting errors with long durations, albeit they tended to overlook short errors (Figs. 2, 3).

**Table 3:** Error detection results on the SAR-RARP50 dataset

Methods	mAP (%)	AUC (%)	Macro-F1 (%)
ResNet-50	31.51	60.75	53.87
TeCNO	35.03	64.58	53.63
MS-TCN	**37.14**	64.58	57.97
MS-TCN++	36.88	**65.10**	**58.97**
Asformer	34.09	64.24	53.42

Higher mAP, AUC, and Macro-F1 mean better model performance. Top results are highlighted in **bold**

## Discussion

This study has successfully demonstrated the feasibility of implementing detailed skill and granular error annotation, using OCHRA, in real robotic surgical video, with promising initial evaluation of deep learning methods in estimations using these input data. Previous applications have predominantly been in the simulated setting [11, 18, 32] with only 3 real cases in laparoscopy (thoracoscopy [33], cholecystectomy [34], gastrectomy [35]) with no reports in robotic surgery.

This research focussed on the feasibility and validity of OCHRA as an error methodology within robotic technical skills assessment, as currently, there are no fully evaluated tools [11]. Most error tools lack the granularity required for annotation purposes, that can be used for AI models. However, arguably more granular suturing assessment tools have also negatively impacted inter-rater reliability, indicating there may be a ‘sweet spot’ between granularity and high IRR [36]. Due to a rapid expansion in the evaluation of machine learning in robotic technical skills assessment, the surgical community must ensure ratings and annotations are highly valid and reliable, which has been achieved across all tools in this study.

OCHRA was chosen as an error annotation tool due to its granular analysis. This group has extensive experience in its application and validation [9, 23, 24, 37–39]. The OCHRA method aims to identify and quantify individual errors from specific subtasks. Errors can broadly be divided into procedural or executional errors [18]. Although it is currently not feasible to apply OCHRA in real-time, it can provide key retrospective, formative insights, with gestures and errors highlighted as the most frequent, and therefore, the most deserving of attention for improvement.

In complex and changing surgical environments, the content of annotation methodology must differ depending on what level of the surgical hierarchy that analysis is performed. Levels of the hierarchy include a whole procedure to multiple steps, tasks, gestures and motions [18, 32]. This study explored the feasability of error annotations at the gesture level in a simple suturing task. Subsequent methodology and AI model evaluation must be further developed based on the hierarchical level and content specific to that task, as other AI methods would not be transferable or externally valid to similar tasks within different surgical procedures or environments.

The vision-based deep learning methods utilised in this study has yielded relatively accurate skill predictions with low mean absolute error values, albeit, demonstrating weak correlation. For error detection, the MS-TCN/MS-TCN++ deep learning model, structured to analyse and aggregate visual temporal information from video sequences, demonstrated the highest performance [29–31] for automated error detection. Although overall accuracy is promising, the Macro-F1 percentages suggest a significant proportion of false negatives/positives. These results underscore the inherent challenges entailed in error detection within real life complex surgical environments, but also promisingly illuminate their initial effectiveness of using vision-based deep learning models for skill and error detection within an operative task.

Thus far, the current literature on AI model application primarily focuses on the open-source JIGSAWS dataset. The results of these studies have been promising, producing accurate predictions of skill assessment scores [11, 40, 41] and events that occur due to unintentional human errors within the simulation setting [18, 42]. Most predict skill or clinical outcomes, which are imperative in the implementation of AI clinically. However, there is an unmet need within research in the prediction of errors [11, 33]. This deserves focussed attention in future research.

Importantly, we intentionally used the suturing task as an exploratory phase but there is a need for further analysis of robotic technical skills beyond simple suturing tasks within only one operation. To ensure validation efforts, reliable annotation is required across multiple specialty tasks and operations. OSATS is the most prevalent tool used in assessing surgical performance in published studies [43], and GEARS is the most evaluated in robotic surgery [11]. Given the need for valid skill ratings in AI development and evaluation, our group would recommend the use of these tools with trained, expert, orientated raters who have consensus sessions to align scoring.

This study exhibited an average of 46 errors per video, which is vastly different when compared to an example of whole procedure error annotations demonstrating a median of 5 per procedure [39]. The observation to be acknowledged is that annotations and the threshold of the level of granularity was intentional to inform deep learning models, and may differ depending on the task. Future studies should address the need to develop and evaluate bespoke deep learning methodologies across a plethora of tasks and operations that can depict relevant skills and errors.

### Limitations

This exploratory study into gesture analysis in the live robotic setting has focused only on simple suturing, paving the way for further research into more complex environments and tasks with more variables such as dissection [44].

Construct validity was not established across assessment tools, most likely due a low number of procedures being carried out by junior registrars. Alternative explanations could be that each group has reached their learning curve plateau on what is considered a simpler task within robotic surgery. Perhaps a difference would be observed in more complex suturing tasks, such as the urethrovesical anastomosis (UVA). DVC was preferred over UVA as the SAR-RARP50 dataset had been pre-annotated for gestures and deemed more useful in the initial study of skills, errors, and AI development.

Most videos were annotated by only one rater due to time constraints. Ideally, errors should be annotated by multiple trained, blinded, expert assessors, representing a challenge for clinical surgeons and future research. Finally, clinical outcomes of the participants were not collected, and therefore, predictive validity could not be investigated.

Automated estimation of OSATS and M-GEARS scores is a challenging task due to our dataset's composition, primarily consisting of highly skilled surgeons. Consequently, scores tend to cluster around the mean, making it difficult for AI models to distinguish subtle differences in execution. Furthermore, the dataset size of 54 data points exacerbates the challenge of attempting a skill assessment regression task. Standard supervised learning approaches occasionally overfit the training set, hindering their ability to generalize to unseen videos. Future directions aim to take advantage of linguistic (e.g. error descriptions), workflow and scene semantics (e.g., surgical gestures, tool segmentation) and multimodal architectures combining both video and robot kinematics to enhance error detection and skill estimation. This is likely to be streamlined with the incorporation of automated performance metrics and event data within robotic systems, such as is expected with the newly announced da Vinci 5 [45]. Additional digital devices and platforms such as the DS1, TouchSurgery™, allow for automated phase segmentation and an annotation platform to enhance research. Another direction is utilizing self-supervised approaches to account for the small dataset size.

Whilst vision-based deep learning models have demonstrated promising performance in error detection, they still face challenges such as relatively high rates of false negatives and false positives, particularly in identifying errors of short duration. This issue stems from the imbalanced distribution of normal and error data, hindering the models' ability to develop robust representations. One potential strategy to mitigate this class imbalance problem is to employ techniques like data augmentation or generative models to augment the error samples. Alternatively, leveraging semi-/unsupervised methods or multimodal learning with textual information, could improve the detection of rare errors. Moreover, our investigation solely explored the viability of deep learning models for binary error detection. Subsequent research endeavours could delve into semantic error detection by incorporating multi-label classification techniques.

## Conclusion

This is the first study to employ OCHRA error methodology and deep learning models within real robotic surgical video. This benchmark evaluation of AI models sets a foundation and promising approach for future advancements in automated skill assessment. It also highlights the inherent challenges for granular error annotation and detection within complex surgical environments. This ultimately demonstrates a step forward towards truly objective robotic technical skills assessment.

## Supplementary Information

Below is the link to the electronic supplementary material.

Supplementary file1 (DOCX 13 kb)
