# Supplementary material for: Deep learning prediction of error and skill in robotic prostatectomy suturing
Source: Surg Endosc. 2024 Oct 21;38(12):7663–71. doi: 10.1007/s00464-024-11341-5 (PMC11614916; doi:10.1007/s00464-024-11341-5)

**Supplementary Material**

**Supplementary Tables:**

Supplementary Table 1: Gesture numbers

Supplementary Table 2: Error coding description

Supplementary Table 3: Surgeon IDs and skill levels

**Supplementary Figures:**

Supplementary Figure 1: Objective Structured Assessment Technical Skills (OSATS) tool

Supplementary Figure 2: Modifiable-Global Evaluative Assessment of Robotic Skills (M-GEARS) tool

Supplementary Figure 3: Suturing task in the JIGSAWS dataset

Supplementary Table 1: Gesture numbers

| Gesture number | Gesture description |
| --- | --- |
| 1 | Picking up needle |
| 2 | Positioning needle tip/positioning needle in the needle driver |
| 3 | Pushing needle through tissue |
| 4 | Pulling needle out of tissue |
| 5 | Tying the Knot |
| 6 | Cutting the suture |
| 7 | Returning/Dropping the needle |
| 8 | Other gestures within suturing |

Supplementary Table 2: Error coding description

| Error code | Description |
| --- | --- |
| 1 | Multiple attempts (end of 1st attempt to end of successful attempt) i.e. multiple piercings, not regrasping with needle held in tissue |
| 2 | Needle drop/slip if in tissue |
| 3 | Instrument(s) out of view |
| 4 | Needle out of view (not binary, depends if dangerous e.g. instrument holding it or suture) |
| 5 | Tissue damage inc. poor/erroneous tissue stabilisation |
| 6 | Incorrect angle grasping needle e.g. not perpendicular (normally) or too far into the jaws |
| 7 | Incorrect position along needle (normally 2/3rds) |
| 8 | Excessive force resulting in damage to tissue or bending of needle |
| 9 | Needle does not follow the curve |
| 10 | Needle entry incorrect angle i.e. not perpendicular |
| 11 | Grasped at needle tip |
| 12 | Suture is loosened |
| 13 | Thread caught in instrument |
| 14 | Knot tied is not square (C/Reverse C) |
| 15 | Inadequate no. of throws |
| 16 | Suture pulled through tissue before tying knot |
| 17 | Incorrect distancing between needle drives i.e. too close/too far |
| 18 | Suture not pulled through between needle drives |
| 19 | Suture entanglement |
| 20 | Fraying the suture |
| 21 | Snapping the suture |
| 22 | Dangerous/poor/incorrect needle disposal |
| 23 | Incorrect/poor camera control inc. blurred/poor view |
| 24 | Incorrect/poor instrument control inc. 3rd arm/non-dominant use, clashing |

Supplementary Table 3: Surgeon ID and skill levels

| Surgeon ID | No of videos | Skill level | Skill level ID |
| --- | --- | --- | --- |
| 1 | 23 | Experienced consultant | 1 |
| 2 | 6 | Early consultant/senior registrar | 2 |
| 3 | 10 | Early consultant/senior registrar | 2 |
| 4 | 6 | Early consultant/senior registrar | 2 |
| 5 | 2 | Experienced consultant | 1 |
| 6 | 3 | Junior registrar | 3 |
| 7 | 1 | Junior registrar | 3 |
| 8 | 1 | Experienced consultant | 1 |

Supplementary Figure 1: Objective Structured Assessment Technical Skills (OSATS) tool


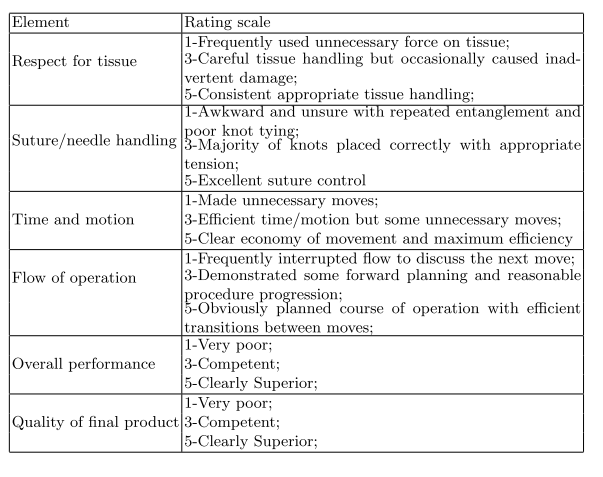


Supplementary Figure 2: Modifiable-Global Evaluative Assessment of Robotic Skills (M-GEARS) tool


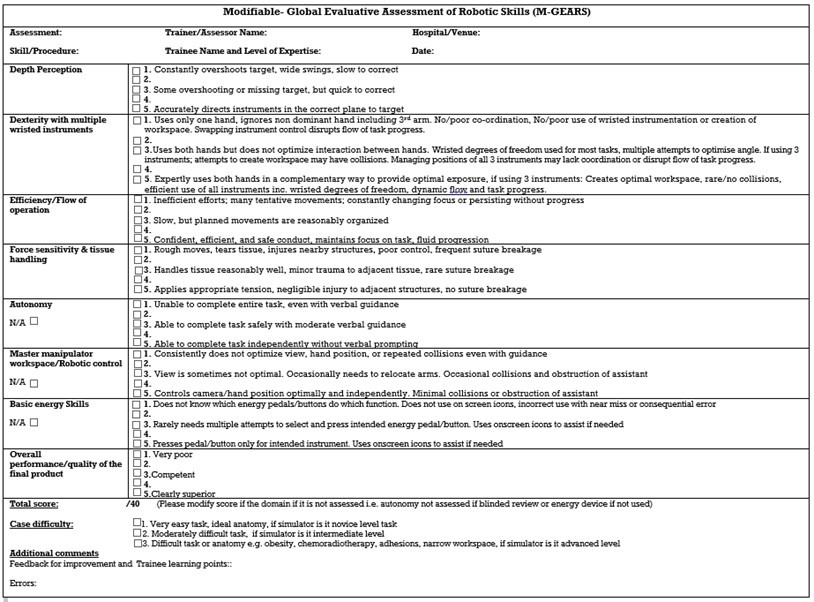


Supplementary Figure 3: Suturing task in the JIGSAWS dataset

#
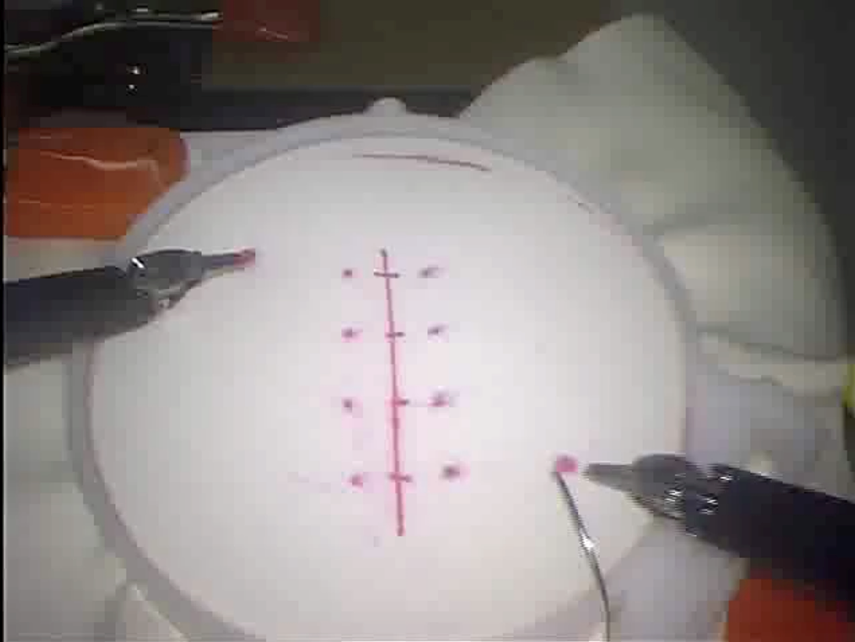

Supplement: Supplementary file 1 — Supplementary file1 (DOCX 13 kb) [file 464_2024_11341_MOESM1_ESM.docx]
